# A Review: Highlighting the Links between Epigenetics, COVID-19 Infection, and Vitamin D

**DOI:** 10.3390/ijms232012292

**Published:** 2022-10-14

**Authors:** Ashmika Foolchand, Siyanda Mazaleni, Terisha Ghazi, Anil A. Chuturgoon

**Affiliations:** Discipline of Medical Biochemistry, School of Laboratory Medicine and Medical Sciences, College of Health Sciences, University of KwaZulu-Natal, Howard College Campus, Durban 4041, South Africa

**Keywords:** SARS-CoV-2, COVID-19, epigenetics, vitamin D

## Abstract

The highly transmittable and infectious COVID-19 remains a major threat worldwide, with the elderly and comorbid individuals being the most vulnerable. While vaccines are currently available, therapeutic drugs will help ease the viral outbreak and prevent serious health outcomes. Epigenetic modifications regulate gene expression through changes in chromatin structure and have been linked to viral pathophysiology. Since epigenetic modifications contribute to the life cycle of the virus and host immune responses to infection, epigenetic drugs are promising treatment targets to ameliorate COVID-19. Deficiency of the multifunctional secosteroid hormone vitamin D is a global health threat. Vitamin D and its receptor function to regulate genes involved in immunity, apoptosis, proliferation, differentiation, and inflammation. Amassed evidence also indicates the biological relations of vitamin D with reduced disease risk, while its receptor can be modulated by epigenetic mechanisms. The immunomodulatory effects of vitamin D suggest a role for vitamin D as a COVID-19 therapeutic agent. Therefore, this review highlights the epigenetic effects on COVID-19 and vitamin D while also proposing a role for vitamin D in COVID-19 infections.

## 1. Introduction

Coronavirus disease 2019 (COVID-19), caused by Severe Acute Respiratory Syndrome Coronavirus 2 (SARS-CoV-2), is still a major focus of research as no definitive treatment has been found. Its rapid spread and mutational rate emphasize the importance of fully understanding the viral structure and its biological function. The SARS-CoV-2 spike protein has been demonstrated to attach to the angiotensin-converting enzyme 2 (ACE2) receptor on the cell surface, which is expressed in the lungs, kidneys, immune cells, vascular endothelia, small intestine epithelial cells, and testes [1,2]. Upon attaching to the host ACE2, viral entry is mediated by an endosome or spike glycoprotein cleavage through host cell proteases such as furin or transmembrane protease serine 2 (TMPRSS2). The viral RNA polymerase then aids replication and transcription of the virus in the cytoplasm, while the host ribosome machinery mediates protein synthesis of the viral RNA. Finally, the virion is assembled in the host’s endoplasmic reticulum-Golgi intermediate complex, enabling the mature virus to integrate into small smooth-walled vesicles and be secreted by the host cells [3].

Upon detection of infection, several host cells attempt to stop or slow viral replication through significant alterations, such as prompting the innate and adaptive immune responses, including cell death in extreme cases. Over time, viruses have co-advanced with their host to develop independent mechanisms to fight or escape the antiviral cellular responses [4]. These mechanisms may induce the reprogramming of the host cell to generate a favorable atmosphere for viral replication, thereby influencing infected cells to become virus-producing factories. The host cell competes against the virus and attempts to constrain it, while the virus counteracts and manipulates the host cell for its own benefit. During this battle, several viral infections initiate epigenetic modifications. Epigenetic modifications control antiviral gene expression as well as the expressions of host cell features employed by the virus for competent replication and transmission [4]. Epigenetic modifications also enable cells to react to environmental deviations and adapt to environmental stimuli via alterations in gene expression profiles.

Over time, amassed research has provided evidence for the role of epigenetics in disease establishment and progression [5,6]. Contrasting mutations, which derange genetic material, epigenetic modifications do not alter the genetic sequence but rather modify the chromatin structure or chemical properties of the nucleic acid [7]. Likewise, many viruses are not capable of altering the genetic sequence. It may alter the epigenome of the host, suggesting that viruses may employ epigenetic mechanisms for infection establishment, transmission, and persistence [8]. Epigenetic alterations are reversible and respond spontaneously to anomalies in the environment [9]. In human diseases, epigenetic modifications include changes in DNA, histone, and RNA patterns [10], which may occur during the infected cells’ response against the disease to return to conventional function.

The heritable epigenetic mark, DNA methylation, occurs at sites rich in CpG dinucleotide repeats, identified as CpG islands. DNA methylation is characterized by the incorporation of a methyl group to the C-5 position of DNA’s cytosine ring, forming 5-methylcytosine. This addition is facilitated by DNA methyltransferase (DNMTs) enzymes in an S-adenosylmethionine-dependent reaction. Studies suggest that five families of DNMTs exist, including DNMT1, DNMT2, DNMT3A, DNMT3B, and DNMT3L [11]. DNA methylation determines the expression of genes via hypomethylation, which results in the activation of gene expression, and hypermethylation, which leads to silenced gene expression [12]. Recent research has linked DNA methylation with *ACE2* gene regulation. Accordingly, increased ACE2 expression within sparsely methylated CpG islands in the lungs, liver, and brain renders these organs more vulnerable to COVID-19 damage [4]. Studies have further deduced that the epigenetic modification footprint appears at various SARS-CoV-2 pathogenesis stages [13]. Additionally, histone modifications also regulate the *ACE2* expression [4]. Post-translational modifications such as methylation of the N-terminal tails of histones occur mainly at the lysine and arginine amino acid residues [14]. Depending on the histone modification, chromatin either loosens to expose transcription factors, facilitating transcription, or condenses to hinder the binding of transcription factors, causing transcriptional repression. Small non-coding RNAs, known as miRNAs, play crucial roles in differentiation, cell growth, apoptosis, signal transduction, and metabolism [15]. The 3′ untranslated regions (3′UTR) of mRNAs are targeted by miRNAs which regulate post-transcriptional gene expressions [16], leading to mRNA degradation or repression of translation [17]. In addition, miRNAs are also implicated in the viral progression and host cell regulation. The level of expression of host miRNAs, which control antiviral activity and viral latency, can be modified by viral infections [18]. Viral miRNAs, produced upon viral invasion, regulate the immune response to the infection [19] and controls their production in the host cell [18]. Viral miRNAs can also promote viral replication by targeting and downregulating specific host genes [18]. Since the COVID-19 pandemic, miRNAs have been proposed to play a role in disease pathophysiology. A better understanding of the miRNA role during COVID-19 infection may aid in discovering novel diagnostic mechanisms, viral combat, and hindering viral entry, replication, and invasion [20].

Vitamin D controls 3% of the human genome [21]. Vitamin D is acquired from the diet and/or synthesized in the skin’s epidermal layer in the presence of UV from sunlight [22]. Vitamin D is widely recognized for its involvement in bone formation; however, emerging evidence has indicated its involvement in other processes, such as immune regulation [23]. Vitamin D targets numerous immune cells such as monocytes, macrophages, dendritic cells, T-lymphocytes, and B-lymphocytes, thus emphasizing its role in both innate and adaptive immunity [23]. These immune cells exhibit vitamin D activating enzymes which convert the inactive vitamin D to its active form, 1,25-dihydroxyvitamin D3, within the immune system; 1,25-dihydroxyvitamin D3 is involved in maintaining immune cell homeostasis [23]. Vitamin D may also aid the host’s defense against viral infections by influencing the expression of toll-like receptors (TLRs) and regulating beta-defensins which cleave the viral membrane [24]. In cases of viral infection, vitamin D impacts the immune system through lysosomal enzyme activation and nitric oxide release, which helps combat infection [25]. Research has indicated the role of vitamin D in downregulating ACE2 receptors, thus suggesting the protective effect of vitamin D against COVID-19 [26]. Several metabolic processes are influenced by vitamin D metabolism, promoting epigenetic modifications that are dysregulated in the disease etiology [27]. Epigenetic regulation of vitamin D occurs primarily via interactions with its receptor (VDR). The discovery of the VDR transcription factor has provided verification for the association of vitamin D in gene expression regulation.

## 2. SARS-CoV-2 Structure

**Figure 1:**
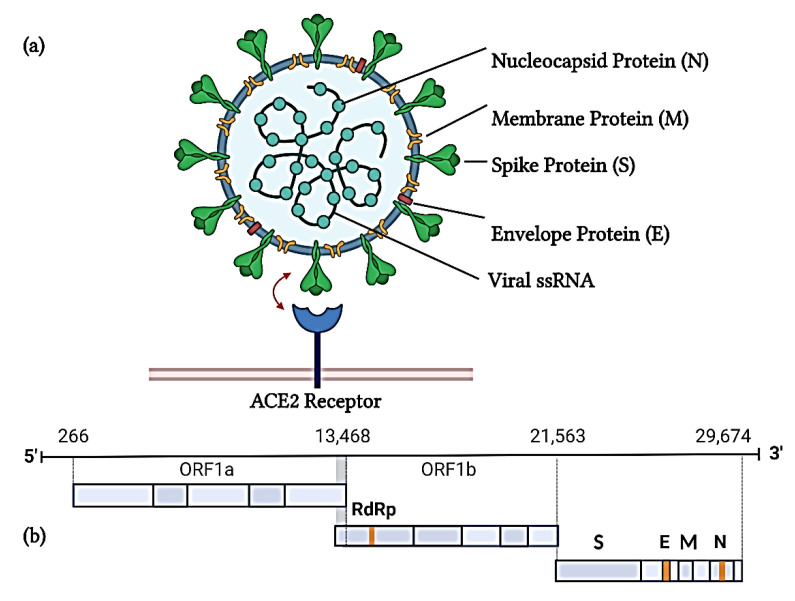
Schematic diagram of the (**a**) SARS-CoV-2 structure and (**b**) its genome. The SARS-CoV-2 genome comprising 29,674 nucleotide base pairs constitutes the nucleocapsid, membrane, spike, and envelope proteins, along with open reading frames (ORF) 1a and 1b. ORF1b contains an RNA-dependent RNA polymerase (RdRp) (Image created on BioRender).

Coronaviruses (CoVs), belonging to the Coronaviridae family, are positive-sense single-stranded, enveloped RNA viruses organized in open-reading frames [28,29]. At 29 kb, SARS-CoV-2 is considered the biggest genome of all RNA viruses [30]. The SARS-CoV-2 genome encodes for a set of non-structural proteins mainly responsible for viral replication and transcription and four structural proteins, namely, the spike, envelope, nucleocapsid, and membrane proteins (Figure 1) [31]. The 5′ region contains the open reading frames (ORF) 1a/b, which is translated to polyproteins pp1a and pp1b that are cleaved by viral proteases into non-structural proteins [31]. The spike protein is a glycoprotein comprising the S1 receptor-binding domain and S2 subunit. While the S1 subunit drives viral attachment to the receptor, the S2 subunit facilitates fusion with the cell membrane [32]. The nucleocapsid protein packages viral RNA into a long helical ribonucleocapsid complex and plays a role in virion assembly [33].The envelope protein assists with virion assembly and ion channel activity [32]. The membrane protein is responsible for new viral particle assembly [32].

## 3. Epigenetics and COVID-19 Infection

It is well known that viruses employ epigenetic processes, particularly CpG methylation, to undergo endocytosis and syncytium formation. During viral progression, the virus fuses with the cell membrane of the host and/or induces host cell-cell fusion. Both these processes promote virus endocytosis, along with the invasion of neighboring cells and innate antiviral immune system evasion [34]. A syncytium, formed during membrane-virus or cell-cell fusion, is generally associated with CoVs, with SARS-CoV-2 being no exemption [35]. Syncytin genes are hypomethylated and highly active in the mammalian placenta, while they are hypermethylated and silenced in other tissues, imposing risks to various diseases [36]. Epigenetics has provided information on developmental biology, heritability, and memory techniques, with research showing more relevance in cancer, immunity, and infectious diseases [37]. Epigenetic research has revealed that DNA and RNA viruses develop antagonizing regulatory roles via host metabolism and gene expression alterations, thereby creating an environment permissive to the spread and replication of the virus [38]. CoVs facilitate epigenetic modifications by antagonizing the host’s presentation of the antigen or by antagonizing the activation of interferon-response genes [39]. In addition, age-related host epigenome modifications may hinder the function and configuration of immune cells, thus influencing viral defenses and the adaptive immunity [40]. This effect of the epigenome may explain the vulnerability of the elderly to SARS-CoV-2 infection [41]. It has also been demonstrated that the *ACE2* gene production rate is epigenetically regulated [42]. Furthermore, based on the methylation patterns of multiple CpG islands, Corley and Ndhlovu [43] showed that the *ACE2* gene functionality is linked to age and gender. The ACE2 receptor, expressed in a range of human organs and tissues, presents the lowest rate of methylation in lung epithelial cells, suggesting that the rate of ACE2 transcription and expression is the highest in the lung tissue [43].

### 3.1. DNA Methylation

Since the COVID-19 outbreak, age, gender, and comorbidities have been considered risk features for increased disease severity and morbidity [11]. Recent research has summarized the association of DNA methylation with *ACE2* gene regulation, suggesting a risk element for COVID-19 illness by the host epigenome [11]. DNA methylation profile analysis of four different lung tissue public databases enabled the assessment of DNA methylation at two *ACE2* CpG sites. It was revealed that age-related DNA methylation in airway epithelial cells occurred in the proximity of the *ACE2* gene transcription start site. Utilizing the Illumina DNA methylation array, samples from diverse biological ages expressed hypomethylation during aging at a CpG (cg085599149) close to the *ACE2* transcription site (TSS200 region). Previous studies have also indicated that aging downregulates global DNA methylation, resulting in differential methylation patterns of aging, inflammatory, and immune response genes [43]. Consistent with epigenetic aging, which results in certain genes becoming more active and other genes becoming less active [44], increased lung tissue ACE2 expression in older people renders them more vulnerable to viral diseases, including COVID-19. In contrast, the *ACE2* gene in organs and tissues of children is typically hypermethylated and thus virtually silenced [12]. This phenomenon may justify the enhanced sensitivity of the elderly to symptomatic SARS-CoV-2 infection in contrast to younger individuals [45].

A DNA methylation array and CHIP methylation genome-wide study demonstrated varied *ACE2* methylation levels in different tissue types. Across three CpG sites (cg03536816, cg04013915, cg08559914), the least *ACE2* gene methylation was observed in lung epithelial cells [43]. Following this, a study revealed that *ACE2* gene hypomethylation was mostly limited to females, suggesting an association between angiotensin II metabolism and hormonal or genetic changes in the chromosome dosage [46]. Additionally, Cai [47] revealed that smokers expressed enhanced *ACE2* gene activity compared with never smokers; this was further confirmed by Leung, Yang [48]. In addition to smoking, testosterone poses another SARS-CoV-2 risk factor as it may be responsible for the increased ACE2 and furin expression in men [12]. Furthermore, the transcriptomic analysis showed no association between *ACE2*, race, age, and gender but showed increased *ACE2* expression in Asian smoker populations [49,50].

An investigation of the DNA methylation profiles from 96 hypertensive patients across five genomic loci of the *ACE2* promoter demonstrated loci- and gender-based variances in methylation patterns, highlighting the consequence of epigenetics on the ACE2 expression [51]. Higher COVID-19 vulnerability has been detected in cancer victims due to DNA methylation at the *ACE2* locus [52]. Investigations on cancer databases showed *ACE2* promoter hypomethylation and enhanced expression of *ACE2* mRNA in colon, pancreas, lung, kidney, rectum, and stomach cancers [53]. Systemic lupus erythematosus (SLE) patients are highly vulnerable to developing symptomatic SARS-CoV-2 infection, which is not a result of their compromised immune system, but rather upregulated ACE2 protein expression in the lung and its associated *ACE2* gene hypomethylation along with significant demethylation of interferon genes [49]. Increased interferon gene expression is associated with severe SLE disease development, distinguished by a cytokine storm [54], which is also a SARS-CoV-2 characteristic [35].

Recently, a study used the Illumina 850k methylation chip to detect DNA methylation expression changes in whole blood samples of COVID-19 patients versus non-infected subjects, thereby introducing novel SARS-CoV-2 infection markers [55]. Upon combining data from the severely infected vs. control group with the mildly infected vs. control group, the researchers identified 35 candidate marker genes that may potentially serve as SARS-CoV-2 infection indicators. Differentially methylated sites from 12 of these candidate genes were located in the promoter region. These genes include *SMG6, CHN2, PDE11A, ATHL1, HECW1, CHST15, MIR510, DCAKD, CPLX2, HYAL1, CRHR2,* and *GNAI2*. In the same study, Zhou, Zhang [55] further investigated the associations between COVID-19 pathogenesis and epigenetic regulation. They found that the severely infected vs. mildly infected group presented the most significant data. GO pathway analysis revealed that differentially methylated gene functions in this cohort largely impacted IL-13, T-helper 2 cell differentiation, neuropeptides, chemokine secretion, and degranulation of mast cells, suggesting a direct link between COVID-19 development and enhanced inflammatory and allergic responses. Additionally, KEG analysis showed that differentially methylated genes are connected to the signaling pathways of phospholipase D, hippo, oxytocin, and sphingolipids, as well as type 1 diabetes, hypertrophic cardiomyopathy, dilated cardiomyopathy and serotonergic synapses [55]. Another study used the Infinium MethylationEPIC array to assess the genome-wide DNA methylation profiles in peripheral blood mononuclear cells [56]. In this study, nine uninfected control subjects were investigated against nine severely ill COVID-19 subjects, nine HIV-1 infected and nine HIV-1/COVID-19 co-infected subjects, including five hospitalized Influenza A or B infected patients. In cells of the severely ill COVID-19 participants, Corley, Pang [56] noted a significant drop in NK cell proportions in comparison with HIV-1 and HIV-1/COVID-19 coinfected participants. The severely ill COVID-19 subjects also presented a significant increase in neutrophils inferred by DNA methylation changes when compared with all other experimental groups. Next, Corley, Pang [56] investigated the genome-wide DNA methylation patterns. In comparison to the controls, 40,904 differentially methylated loci were identified. Severely ill COVID-19 individuals also presented 26,733 differentially methylated loci against influenza, while 51,728 differentially methylated loci were identified against HIV-1. Notably, significant hypermethylation was discovered in type 1 IFN response genes regulatory regions among the severely ill COVID-19 subjects with differentially methylated loci. This finding supports the idea of host IFN responses being repressed by SARS-CoV-2 as the *IFITM1* and *ISG20* first-line antiviral defense genes were among the impacted genes [56]. In contrast, regulatory regions of immune-inflammatory and cytokine genes, including the *NLRP3* inflammasome and *MX1* antiviral genes, displayed hypomethylation corresponding with severe COVID-19. Irregular DNA methylation patterns were also observed in relation to severely ill COVID-19 subjects’ *ACE2* expression. Lastly, Corley, Pang [56] discovered a significantly enhanced epigenetic age in severe COVID-19 when compared with the control and influenza-infected groups. The severe COVID-19 group also demonstrated increased mortality risk in contrast to the control, HIV-1, and HIV-1/COVID-19 coinfected groups [56]. In blood samples of 124 hospitalized COVID-19-positive and negative patients, Balnis, Madrid [57] conducted a genome-wide analysis, utilizing the Infinium Human MEthylationEPIC Beadchip, on circulating CpG methylation and compared it to previous, pre-pandemic data collected of 39 healthy controls. [57] found no significant differences in levels of global mean methylation among the pre-pandemic healthy controls and COVID-19 patients. Locus-specific DNA methylation levels were then investigated, revealing more hypomethylated differentially methylated regions (DMR) than hypermethylated DMRs. Gene ontology analysis, investigating the links between DMR-associated genes, then revealed significantly enhanced immune and defense responses, leukocyte activity, components of type 1 interferon signaling pathway, and replication of the viral genome. Furthermore, significant links between DMR-associated genes and autoimmune diseases, such as lupus erythematosus and rheumatoid arthritis, were revealed in COVID-19 patients via disease ontology analysis [57]. Coinciding with this finding, epigenome-wide analysis of peripheral blood samples conducted by Konigsberg, Barnes [58] discovered significantly enhanced pathways and genes associated with interferon signaling and viral responses.

### 3.2. Histone Modifications

Severe COVID-19 cases have been correlated with high lung ACE2 expression in patients with comorbidities. Experimental and network analysis from 700 human lung transcriptomes with high *ACE2* expression and underlying conditions identified histone acetyltransferase 1 (HAT1), histone deacetylase 2 (HDAC2), and lysine demethylase 5B (KDM5B) as prospective regulators of *ACE2* [45]. HDAC2 plays a role in immune responses against viruses [59] and contains a cleavage site that is possibly targeted by the SARS-CoV-2 non-structural protein NSP5. Therefore, interactions with NSP5 may inhibit the HDAC2 nuclear localization [4]. Pathway enrichment evaluation disclosed that *ACE2*-related genes were controlled by KDM5B as well as specific histone methylation and acetylation marks, including mono-methylation (me) and trimethylation (me3) of lysine 4 (K4) on histone H3 and histone H3 acetylation of lysine 27 (H3K27ac). KDM5B regulates chromatin accessibility by repressing active chromatin marks, including di- and trimethylation of H3K4, which plays a role in DNA repair and gene transcription. Additionally, breast cancer patients demonstrated that KDM5B inhibition triggered an interferon response, resulting in resistance to DNA and RNA viral infections, thereby proposing a role for KDM5B as a COVID-19 prevention target [45].

Under cell energy stress conditions, ACE2 may also be regulated by the NAD-dependent histone deacetylase sirtuin 1 (SIRT1), which was enhanced in comorbid COVID-19 patients’ lungs [45]. Interferons mediate antiviral responses and induce pathogen-driven immune responses by interferon-stimulated gene (ISG) inactivation [60]. During SARS-CoV infections, ISG promoter regions displayed more active H3K4me marks than H3K27me3 repressive marks, thus favoring decondensed chromatin and stimulating active transcription and expression of ISGs [61,62]. Li, Li [63] investigated the impact of histone modifications on *ACE2* gene expression via the histone methyltransferase enhancer of zeste homolog 2 (EZH2), which catalyzes H3K27me3. In human embryonic EZH2 knockout cells, *ACE2* levels were increased, indicating increased transcriptional regulation. ChIP-seq analysis at the ACE2 promotor region in EZH2 deficient cells indicated decreased levels of H3K27me3, along with enhanced levels of H3K27 acetylation [63]. The researchers further examined the effects of H3K4me1 and H3K4me3 in EZH2 knockout cells but found no significant changes in the ACE2 promoter region [63].

A study of 117 COVID-19-positive patients displayed increased histone H3 levels upon ICU admittance, while 50% of patients presented detectable histone H3 levels at least once throughout their ICU stay [64]. During ICU admission, any detection of histone H3 was correlated with an escalated prevalence of thromboembolic events and secondary infections [64]. Furthermore, non-cleaved histone H3 was also linked to an elevated prevalence in thromboembolic events, while full or partial proteolytic cleavage of histone H3 was only discovered in 23% of COVID-19 patients [64]. A study among individuals simultaneously affected by acute respiratory distress syndrome and SARS-CoV-2 during their initial ventilation period showed increased circulating histone-associated DNA complexes in the plasma of COVID-19 patient samples, compared with healthy controls [65]. In this study, Bouchard, Colovos [65] also observed altered fibrin formation along with elevated endothelial-dependent thrombin production, hypothesizing its coagulation role in COVID-19 patients.

Moreover, another epigenetic modification, citrullination of histone 3 (Cit-H3), has been observed in COVID-19 patients. Cit-H3 involves the conversion of arginine residues on histones to citrulline, which loosens the chromatin structure and enhances transcription factor accessibility [66]. Elevated Cit-H3 levels in COVID-19 victims were linked to increased interleukin-8, leukocytes, and granulocyte counts [67]. Cit-H3 is also a neutrophil extracellular traps marker (NETs) that indicates the immune response to infection [68]. The short life span of neutrophils is linked to NETs or NETosis, where activation of cell death mechanisms may be a major contributor to enhanced Cit-H3 in COVID-19 [69]. Zuo, Yalavarthi [69] suggested that irregular platelet counts may be credible to NETs in COVID-19-infected people upon observing a positive correlation between platelet counts and Cit-H3 levels. In COVID-19 patients, tissue damage and sepsis may also increase NETosis. Symptoms of sepsis in organs and circulation, such as organ damage, cytokine storm, and increased NETs, predict worse outcomes in COVID-19 patients in which circulating neutrophil levels are increased [70,71]. Further, plasma samples of COVID-19 patients with sepsis presented increased Cit-H3 levels [72]. Histone citrullination has also been reported in other human diseases such as cancer, autoimmune diseases, and thrombosis, which are also major threats for severe COVID-19-infected patients [4].

### 3.3. miRNAs

Over time, miRNAs have gained popularity as a novel tool for medical therapies [73] and have been recognized as regulators of viral infections [16]. Viral miRNAs encoded by SARS-CoV-2 can target multiple host genes. A study predicted 3377 target human genes as distinctive targets of 170 mature miRNAs from SAR-CoV-2. The spike protein and *open reading frames* (ORF) *1ab* are targeted by 67 and 369 different miRNAs, respectively, while 10 identified miRNAs contain binding sites across the SARS-CoV-2 genome [20]. Farr, Rootes [74] analyzed the circulating miRNA profiles in the plasma of COVID-19 patients. Differential expression in COVID-19 patients was observed in 50 miRNAs, of which 30 miRNAs were downregulated while 20 miRNAs were upregulated. Among these, miR-31-5p, miR-3125, and miR-4742-3p were the most upregulated candidates, while miR-1275, miR-3617-5p, and miR-500b-3p were the most downregulated of all miRNAs. Anti-inflammatory miR-766-3p showed the greatest statistically significant change. Farr, Rootes [74] then determined whether miRNA profiles of COVID-19 patients could independently identify SARS-CoV-2 during early infection stages. Logistical regression analysis revealed that the measurement of mi-R195-5p showed a 90% accuracy, 95% precision, and 72% recall for COVID-19 identification, while a combination of miR-423-5p, miR-23a-3p, and miR-195-5p received 99.9% accuracy, 99.8% precision and 99.9% recall [74].

Another study carried out by de Gonzalo-Calvo, Benítez [17] also analyzed miRNA profiles of hospitalized COVID-19 patients and investigated its potential as a biomarker. Among the circulating miRNAs of ward and ICU patients, 10 out of 41 miRNAs showed differential expression. ICU patients expressed an upregulation of miR-27a-3p, miR-199a-5p, miR-148a-3p, miR-491-5p and miR-27b-3p in contrast to miR-486-5p, miR-16-5p, miR-150-5p, miR-451a and miR-92a-3p which were downregulated [17]. LASSO regression further identified miR-451a, miR-148a-3p, and miR-486-5p as signatures linked to ICU admission. Next, de Gonzalo-Calvo, Benítez [17] examined the impact of medicines commonly administered to patients for hospitalized COVID-19 treatment on circulating miRNAs. Results revealed significant effects of antibiotics on miR-150-5p, miR-16-5p, and miR-92a-3p, while corticoid and hydroxychloroquine only influenced miR-92a-3p and miR-150-5p, respectively. Additionally, the total duration of ICU admission was revealed to have an inverse correlation with miR-214-3p, miR-150-5p, miR-93-5p, miR-92a-3p, and miR16-5p, upon investigating the link between the length of ICU stay and circulating miRNAs [17].

A study by Arisan, Dart [75] determined 7 important miRNAs (miR-1468-5p, miR-3691-3p, miR-1307-3p, miR-3934-3p, miR-3611, miR-5197 and miR-8066) with key links to host response and viral pathogenicity. In the peripheral blood of COVID-19 patients, the elevated miRNAs included miR-618, miR-16-2-3p, and miR-6501-5p. In contrast, these patients presented reduced expressions of miR183-5p, miR-144-3p, and miR-627-5p, which correlated with impaired immune function via differential miRNA expression [76].

In silico analysis by Fulzele, Sahay [77] determined 873 common miRNAs targeting the COVID-19 genome among 29 isolates, while 315 miRNAs were unique for COVID-19, and 19 of the 29 isolates had identical miRNA targets. In addition 6 miRNAs were found to be unique to the Netherlands (hsa-let-7b-3p, hsa-miR-300, hsa-miR-4666a-3p, hsa-let-7a-3p, hsa-miR-381-3p, and hsa-miR-98-3p) with Australia (hsa-miR-892c-5p, hsa-miR-5088-5p, hsa-miR-3677-5p and hsa-miR-9900) and Wuhan (hsa-miR-4474-3p, hsa-miR-6762-3p, hsa-miR-10401-5p and hsa-miR-4304) presenting 4 unique miRNAs each [77].

Research also speculates that decreased miR-146 levels in diabetic, hypertensive, and obese patients may enhance the SARS-CoV-2 infection susceptibility of these individuals [78]. Recently a study revealed elevated miR-146a-5p levels in the serum of COVID-19 patients responding to tocilizumab treatment. Post-treatment, patients who did not respond to tocilizumab presented reduced miR-146a-5p serum levels [79]. In human alveolar and bronchial epithelial cells, transcriptomic analysis displayed that uncontrolled inflammation during COVID-19 may be attributed to dysregulated miR-1207-5p via overexpression of the CSF1 gene, which promotes recruitment and activation of macrophages [80]. Hsa-miR-1307-3p, highly expressed in the lung, plays a suppressive role in proliferation, exocytosis, endocytosis, and diabetes signaling while possessing a high affinity for the 3′UTR of the SARS-CoV-2 genome. Thus, the binding of hsa-miRNA-1307-3p to the 3′UTR of the viral genome may aid in viral replication inhibition [81].

Host miRNAs control vital protein expression in SARS-CoV-2 invasion. Viral spike protein synthesis was seen to be regulated by hsa-miR-5197-3p and hsa-miR-8066, whereas hsa-miR-3934-3p present SARS-CoV-2 binding sites [20]. Studies have also documented the host miRNAs which target ACE2 expression. miR-1246 increases ACE2 expression, which is linked to ARDS [82]. In human cardiomyocytes, increased miR-200c expression leads to decreased ACE2 expression [83]. Research also indicates that ACE2 expression is known to be decreased by miR-421 and miR-143 [84]. In addition, Widiasta, Sribudiani [85] also indicate the role of miR-18 as a regulator of ACE2 expression in the kidney and a potential COVID-19 therapeutic agent.

## 4. Vitamin D Regulation

**Figure 2:**
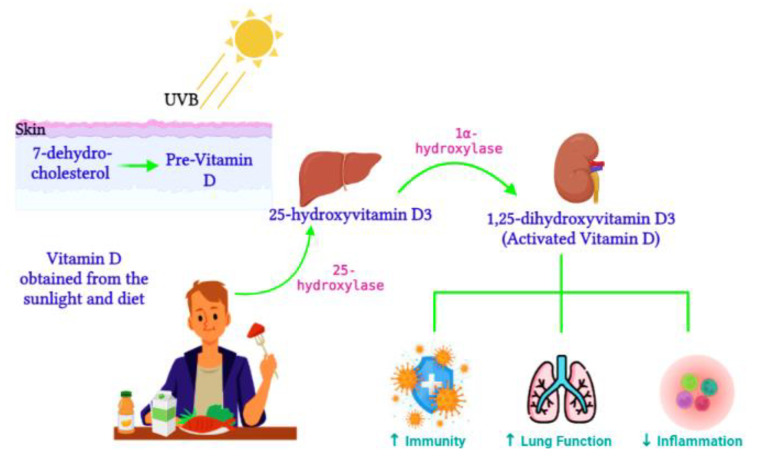
Vitamin D metabolism. During sunlight exposure, UVB radiation converts 7-dehydrocholesterol to pre-vitamin D in the skin. Vitamin D acquired from the diet t is converted by 25-hydroxylase to 25-hydroxyvitamin D3 in the liver. Activated vitamin D, 1,25-dihydroxyvitamin D3, is then synthesized in the kidney. Activated vitamin D improves immunity and lung function and reduces inflammation (Image created on Biorender.com).

Active vitamin D, 1,25-dihydroxyvitamin D3, is synthesized from the 25-hydroxyvitamin D3 precursor via the 1α-hydroxylase enzyme (CYP27B1) (Figure 2). CYP27B1 is expressed in epithelia which forms the main barrier between the body and its environment. Upon invading pathogens, epithelia respond via its own innate immune system, activating macrophages and dendritic cells to recruit T cells and neutrophils to the infection site [86]. Therefore, during vitamin D insufficiency, reduced availability of the vitamin D precursor would impair active vitamin D production and subsequently alter the innate immune function [87]. Over time, vitamin D deficiency has surged worldwide [88] and has been connected to impaired lung function, enhanced inflammation, and poor immunity [89]. Ligand binding stimulates the translocation of the VDR from the cytosol to the nucleus, where the VDR heterodimerizes with the retinoid X receptor (RXR) to form the VDR/RXR complex. This complex attaches to specific genomic sequences on the promoter region of target genes, known as vitamin D response elements, thus activating or suppressing gene transcription on promoters by recruiting transcription factors and co-regulatory molecules [90]. Active vitamin D is vital for immune regulation. In contrast, vitamin D deficiency was previously associated with the pathogenesis of chronic lung diseases such as asthma and chronic obstructive pulmonary disease in populations exposed to airborne particulates [91]. Vitamin D enhances innate immunity via the antiviral peptide secretion [92], which enhances mucosal defenses. Dietary vitamin D controls genes involved in cell apoptosis, proliferation, differentiation, immune responses, and inflammation [93]. Smoking has also been identified to alter vitamin D metabolism in the lungs [94]. Furthermore, vitamin D consumption was shown to improve the innate immune response against respiratory pathogens and improve the respiratory health of vitamin D-deficient individuals [95].

### 4.1. The Impact of Vitamin D on Epigenetics and Gene Expression

Vitamin D was originally described as regulating calcium homeostasis; however, it now has crucial functions in various biological processes and diseases. Vitamin D metabolism promotes epigenetic modifications by influencing metabolic factors. While other vitamins (vitamin B12) and nutrients act as direct donors of methyl groups or inhibitors (e.g., resveratrol) of DNA promoter methylation [96], the epigenetic mechanisms of vitamin D are induced mainly by its receptor VDR. This could also involve the expression of CYP27A1 and CYP27B1, which are key enzymes associated with converting Vitamin D3 to pre-hormones calcitriol and calcidiol and the CYP24A1 inactivating enzyme [97]. Therefore, calcitriol and calcidiol both mediate vitamin D signaling to the VDR, which often builds dimers with retinoid X receptors [98] and attaches to vitamin D response elements, thus influencing gene transcription. During transcription, the VDR/RXR dimers network with HATs, introducing acetyl groups to the nucleosomes and opening chromatin, thus activating the transcription [99]. It is suggested that VDR promoter methylation may also establish a calcitriol insensitivity [100]. VDR-mediated epigenetic regulation of microRNAs [101] or tumor suppressor genes [102] control the response of target promoter genes. Gene expression studies indicate that drugs under epigenetic activation potentially reverse calcitriol insensitivity. Genome-wide analysis revealed that VDRs typically attach at the loci of open chromatin, and upon 1,25-dihydroxyvitamin D3 liganded VDR treatment, chromatin accessibility of these loci is increased [103]. The mechanisms of liganded VDR activity depend on the binding and activity of histone acetyltransferases and methyltransferases [104]. Epigenetic corruption of VDR signaling leads to poor 1,25-dihydroxyvitamin D3 responsiveness, which may occur via promoter methylation of important vitamin D genes or by a skewed build-up of VDR-associated co-repressors, favorably at anti-proliferative target gene promoters [105]. In addition, microarray studies revealed VDR reactivation mediated by HDAC inhibitor trichostatin A and calcitriol upregulated a group of repressed gene targets linked to apoptosis and proliferation [106]. In dendritic cells, ligand-dependent HDAC-containing complexes attach to VDR and the transcription factor *relB* promoter. Experimental analysis reveals that HDAC3 negatively regulates lipopolysaccharide-stimulated *relB*, leading to the detachment of VDR/HDAC3 from the *relB* promoter and thus highlighting the significance of chromatin remodeling induced by vitamin D in the regulation of dendritic cell activity [107]. VDR has also been demonstrated to interact with Forkhead box O (FOXO) proteins, its regulator SIRT1 and 1,25-dihydroxyvitamin D, along with stimulation of SIRT1- and phosphatase-dependent dephosphorylation and FOXO protein activation [108]. VDR also impedes NF-κβ activity via SIRT1 and 1,25-dihydroxyvitamin D signaling, suggesting the role of 1,25-dihydroxyvitamin D-induced NF-κβ deacetylation [109]. Furthermore, research has shown that VDR promotes p21^(waf1/cip1)^ expression and cell cycle arrest; this was related to a VDR-mediated feed-forward loop and interplay of H3K9ac and H3K27me3 [102].

### 4.2. The Role of Vitamin D in COVID-19 Infections

The initial link between vitamin D and the immune system was brought about by the synthesis of active vitamin D from its precursor by antigen-presenting cells such as dendritic cells and macrophages. Active vitamin D, produced in the lungs, is vital for pulmonary immune responses. In airway epithelium, vitamin D controls VDR-induced gene expression to identify and eliminate pathogens through TLR co-repressor CD14 and antimicrobial peptide mechanisms [110]. Additionally, in airway epithelium, NF-κβ-induced cytokine and chemokine expressions are modulated by 1,25-dihydroxyvitamin D during viral infections [111]. To decrease the hazard of common colds, vitamin D utilizes three pathways: adaptive immunity, physical barrier, and natural cellular immunity. To minimize COVID-19 infection and mortality risk, vitamin D possibly functions in maintaining gap and cell junctions, reducing the cytokine storm, enhancing cellular immunity, and regulating adaptive immunity via responses of T helper cell type 1 and inducing T cells [112]. In terms of COVID-19 infection, vitamin D promotes antiviral immunity by inducing defensins and cathelicidin that hinder viral entry and impairs viral replication [113]. Vitamin D also promotes autophagy, an essential homeostasis mechanism involving the encapsulation of misfolded proteins and impaired organelles. Encapsulation of viral particles by autophagy packages these particles for lysosomal degradation, followed by antigen presentation and adaptive antiviral immune responses [114]. Autophagy stimulation is a vital vitamin D cellular response as active vitamin D and its precursor both enhance the expression of the autophagy marker LC3 [115]. Importantly, the consequences of vitamin D on autophagy are closely associated with apoptosis, potentially aiding viral replication, suggesting a role for vitamin D in autophagy and apoptosis equilibrium for optimal antiviral responses against infections [116].

An evaluation of 20 hospitalized COVID-19 patients revealed a vitamin D deficiency in 75% of the overall cohort, with 85% of the patients requiring ICU treatment [117]. In Europe, an analysis of vitamin D status revealed that a deficit of vitamin D was prominent in countries with increased rates of COVID-19 death and infection [118]. Additionally, preliminary data from the US documented a strong correlation between vitamin D insufficiency with poor outcomes and death [119], while Maghbooli, Sahraian [120] reported a statistically significant decrease in unconsciousness and hypoxia in patients with vitamin D sufficiency A retrospective study of 212 COVID-19 patients from three different South Asian hospitals revealed significantly different mean vitamin D levels among mild, ordinary, severe and critical COVID-19 cases [121]. In contrast, Indian patients showed no correlation between baseline vitamin D levels, mortality, length of hospital stay, or need for ventilation [122].

Randomized control trials have shown a reduction in the risk of respiratory diseases following vitamin D supplementation [123]. In Singapore, an observational study comprising 43 cases reported that fewer COVID-19 patients required oxygen treatment following a combined supplementation of vitamin D, B12, and magnesium [124]. Another study showed that vitamin D supplementation led to only two COVID-19 patients requiring ventilation compared with nine patients in the control group. The vitamin D-supplemented group only had one mortality compared with six in the control group, while the patients supplemented with vitamin D also presented a lower hospital admittance period [125].

A retrospective Indonesian study revealed that older males with comorbidities were at a higher risk of COVID-19 death [126]. Furthermore, in Italy, reduced 25-hydroxyvitamin D levels were observed in hospitalized COVID-19 patients [127]. Significantly lower serum 25-hydroxyvitamin D was additionally observed among Chinese patients [128]. Upon investigating the association between serum 25-hydroxyvitamin D and the extent of pulmonary and clinical involvement in COVID-19 patients, Abrishami, Dalili [129] found that elevated 25-hydroxyvitamin D levels were linked to reduced lung involvement and risk of mortality. Maghbooli, Sahraian [120] also revealed that of 206 patients 40 years and older, 20% who succumbed to COVID-19 presented less than 30 ng/mL blood levels of 25-hydroxyvitamin D, while only 9.7% with at least 30 ng/mL 25-hydroxyvitamin D blood levels died. In children with SARS-CoV-2 Omicron subvariant BA.2, Peng, Huang [130] highlighted that a 25-hydroxyvitamin D serum concentration of at least 30 ng/mL may improve clinical outcomes by promoting viral clearance early in the disease course and ameliorating pneumonia lesions. Other research has shown that serum 25-hydroxyvitamin D is inversely linked to IL-6 and proinflammatory cytokines [131,132].

## 5. Potential COVID-19 Therapeutic Targets

The broad ACE2 receptor distribution across several cell types imposes challenges for the therapeutic targets against COVID-19. Besides ACE2, another critical potential regulator of SARS-CoV-2 infection is the TMPRSS2 cellular membrane serine protease. Upon infection and SARS-CoV-2 attachment to the cell surface, the spike protein is primed by TMPRSS2, allowing S1/S2 cleavage and fusion of the viral and cellular membranes at the S2 site [133,134]. Targeting the SARS-CoV-2 cellular entry is a promising treatment method; hence researchers have focused on developing neutralizing antibodies that inhibit the binding interactions of ACE2 with the spike protein [135]. Other than neutralizing antibodies against the viral spike protein, many chemical compounds are being evaluated to obstruct TMPRSS2 and ACE2 protein activity and binding capacity. The clinically approved camostat mesylate is a serine protease inhibitor that has demonstrated repressive effects against TMPRSS2 activity, suggesting its potential as an effective COVID-19 infection treatment [3]. Additionally, miRNAs associated with ACE2 and/or TMPRSS2 expression may also serve as potential targets for COVID-19 therapeutic applications.

The vital role of epigenetic regulations in COVID-19 mechanisms suggests the potential of epigenetic enzymes as therapeutic targets. Two clinical trials are in progress to assess epigenetic mechanisms as a COVID-19 treatment strategy; The first ongoing trial (NCT04403386) aims to investigate the impact of smokers versus nonsmokers on the immune system, prior and post-COVID-19 infection. This study will compare biomarkers for smoking exposure with participants’ immune cell profiles and their transcriptional and DNA methylation profiles of the immune cells to determine whether smokers are at greater risk of COVID-19-induced morbidity and mortality [4]. The second ongoing trial (NCT04411563) involved the assessment of the DNA methylation status of *ACE2*, *TMPRSS2,* and *PARP* (interleukin activator in the cytokine storm) genes in an attempt to understand the link between epigenetic modifications (DNA methylation and microRNAs) and COVID-19 severity in the presence or absence of severe acute respiratory syndrome and pneumonia, in Turkish patients [4]. Currently, combined epigenetic and antiviral drug therapy is valuable for impairing viral replication and regulating host immune response [136]. ACE2 expression has previously been revealed to be regulated by DNA methylation and histone modifications, thereby proposing epigenetic enzymes (DNMT1, HAT1, HDAC2, and KDM5B) as probable targets to regulate host immune responses [11,53]. In this context, inhibitors of DNMT1, HAT1, and HDAC2, namely azacitidine, anacardic acid, and valproic acid, respectively, may be repurposed against CoV infections [59,137]. The cytokine storm is a key contributor to COVID-19 mortality and is characterized by the uncontrolled over-production of inflammatory markers to which the elderly, immunocompromised, and chronically ill patients are more susceptible. Pro-inflammatory cytokine and chemokine expressions are enhanced with the demethylation of NF-kβ, IFN-related genes, and important cytokine genes. Therefore, reducing the concentrations of plasma IL-6 along with the epigenetic regulation of *ACE2* may provide a COVID-19 prevention and treatment target [49]. The nucleoside-based DNMT inhibitor, decitabine or 5-aza-2-deoxycytidine, is often utilized to obstruct DNA methylation in macrophages, therefore suppressing IFN and inflammatory responses [136]. In fact, decitabine was recently included in an ongoing COVID-19 Pneumonia-ARDS treatment clinical trial (NCT04482621). Potential preventive natural compounds such as the DNA methyltransferase inhibitor curcumin, sulforaphane, and 8-hydroxyquinoline may induce epigenetic silencing of *ACE2* towards SARS-CoV-2 infection [13]. Particularly, curcumin is of great interest, owing to its ferritin-reducing effects [138] and considering that severe SARS-CoV-2 patients presenting increased ferritin levels suffer worse outcomes [139]. Many antiviral drugs have been and are still under investigation for COVID-19 treatment strategies. These antiviral drugs include the influenza Favipiravir and Umifenovir drugs and the HIV Lopinavir/ritonavir drug, either alone or in combination with antimalarial chloroquine or hydroxychloroquine, convalescent plasma, vitamin C and D supplementation, mesenchymal stem cell (MSC) and MSC-derived exosomes and Chinese traditional medicines [59,137].

A recent Mass Spec-based assay revealed that the SARS-CoV-2 transmembrane envelope protein binds to transcription factors Bromodomain (BRD) Containing 2 (BRD2) and BRD4 of the host cell. The BRD family, along with the bromodomain and extra terminal domain family (BET), are epigenetic readers which identify acetylated chromatin. BRD4 is a major regulator of gene expression. BRD4 inhibition displays significant antiviral effects against various DNA and RNA viruses by inducing an innate immune response, enhancing DNA damage response, facilitating blockage of viral attachment, reducing viral replication, and arresting cell cycle without apoptosis [59,140]. BRD2 and BRD4 bind to the N-terminal of acetylated histones, thereby regulating the transcription of the target genes. Upon infection, these BRDs interact with the virus envelope proteins’ C-terminal, which mimics the H3 histone tail N-part. As a result, BET inhibitors are suggested to restrict these interactions and disturb the viral and host cell transcriptional mechanisms [59]. Preclinical BRD and extra-terminal protein inhibitor candidates, JQ-1 and dBET6, efficiently avert SARS-CoV-2 genome replication by disturbing the communication between BRD4 and the virus transmembrane envelope protein [59,140]. HDACs represent the main epigenetic agent for viral infection therapies [141]. Previously, resveratrol and SIRT1 have been labeled as antiviral effectors [142]. Resveratrol targets viral infection regulation by modulating the activity of SIRTs (SIRT1, 2, 3, 5), particularly via SIRT1, protein kinase C, activator protein 1, 5′ AMP-activated protein kinase (AMPK), nuclear factor kappa β (NF-kβ), p53, early growth regulator 1 (EGR-1), sterol regulatory element-binding protein 1 (SREBP-1) and DNMT1 [142]. Several viral non-structural proteins that play a role in maturation, transcription, and replication are under HDAC regulation, suggesting that antivirals combined with suberanilohydroxamic acid and vorinostat HDAC inhibitors may be useful against infections [143]. Another potential target, the polycomb repressive complex 2 (PRC2), induces transcription suppression through increased H3K27me3 at specific IFN-stimulated genes. Advanced clinical trials for PRC2 pharmacological inhibitors in cancer treatment are underway, which could be repurposed for COVID-19 treatment [144]. It has also been observed that β-glucan-mediated trained immunity controls some epigenetic alterations and could thus represent a suitable COVID-19 therapeutic target [145].

Several studies have recognized miR-143 and miR-421 as negative ACE2 regulators, while miR-27a/b and miR-145 expressions are said to positively correspond with ACE2 levels [146]. It is also suggested that miR-5197-3p, miR-2113, miR-429, miR-300, miR-200-3p, miR-106b-5p, miR-130a-39 and miR-141-3p may serve as potential ACE2 regulators which may be targeted to fight COVID-19 [146]. An additional potential miRNA treatment strategy includes the inhibition of mRNA encoding the SARS-CoV-2 spike protein. Gallicano, Casey [147] found that miR-510-3p and miR-624-5p targeted the ORF of viral spike RNA, with miR-624-5p being more effective in suppressing the spike RNA. Bioinformatic analysis by Khan, Sany [148] identified three host miRNAs (hsa-miR-323a-5p, hsa-miR-20b-5p, and hsa-miR-17-5p) which could regulate viral infections via host immune responses. Furthermore, Zhou, Zhou [149] suggests a role for miR-2911 in honeysuckle decoction in SARS-CoV-2 replication inhibition.

In vitro data presented by Glinsky [150] suggested that vitamin D and quercetin halt ACE2 and furin expression, presenting themselves as interesting preventive agents for SARS-CoV-2 amelioration. In addition, Ilie, Stefanescu [151] revealed a negative correlation between average vitamin D levels and SARS-CoV-2 mortality rate in European countries, supporting the preliminary recommendations of vitamin D as a SARS-CoV-2 treatment intervention. Vitamin D also seems to influence β-defensin 2, an innate antibacterial element [152]. β-defensin 2 induces antiviral chemokines and cytokines responsible for monocyte/macrophage, T cells, neutrophils, and natural killer cells recruitment, thus contributing to host defense [153].

## 6. Conclusions

COVID-19 is a difficult disease to combat due to its rapid spread and high mutational rate. Despite the steady rollout of the COVID-19 vaccines, presently, no clinically accepted antiviral drug is available for the effective therapy of COVID-19. Combination therapies may effectively reduce drug resistance, viral replication, and toxicity through synergistic interactions. Combining antiviral drugs with epigenetic remedies such as DNMT/HDAC inhibitors may be a credible alternative; however, pre-clinical and clinical trials are required to validate this. Targeting miRNAs associated with immune responses and regulating ACE2, TMPRSS2, and spike protein expressions may also provide an effective treatment approach against COVID-19. Several studies have documented the incidence of vitamin D deficiency in severe COVID-19 patients. Although the immunomodulatory effects of vitamin D and its function in immune homeostasis have been highlighted, well-designed trials are mandatory to confirm the plausible protective role of vitamin D in COVID-19 infections.
